# Supplementary material for: Hepatic Histopathological Benefit, Microbial Cost: Oral Vancomycin Mitigates Non-Alcoholic Fatty Liver Disease While Disrupting the Cecal Microbiota
Source: Int J Mol Sci. 2025 Sep 4;26(17):8616. doi: 10.3390/ijms26178616 (PMC12429848; doi:10.3390/ijms26178616)
Supplement: Supplementary file 1 [file ijms-26-08616-s001.zip › ijms-3838057-supplementary.pdf]

Supplementary Figures

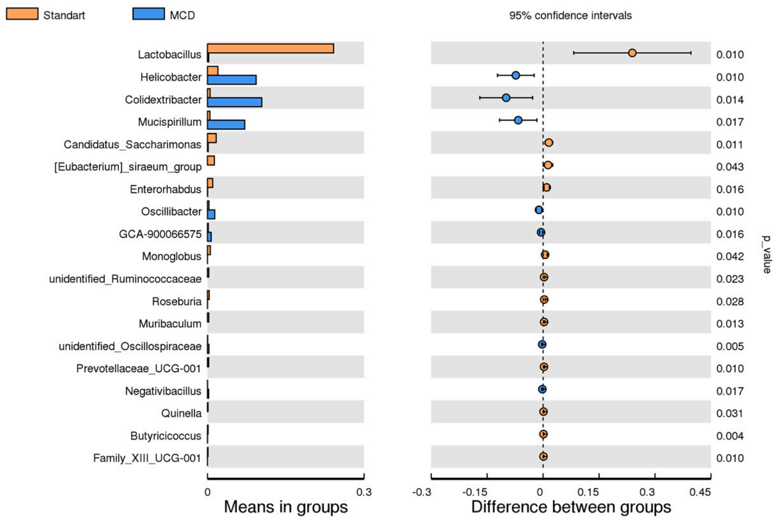

(**a**)

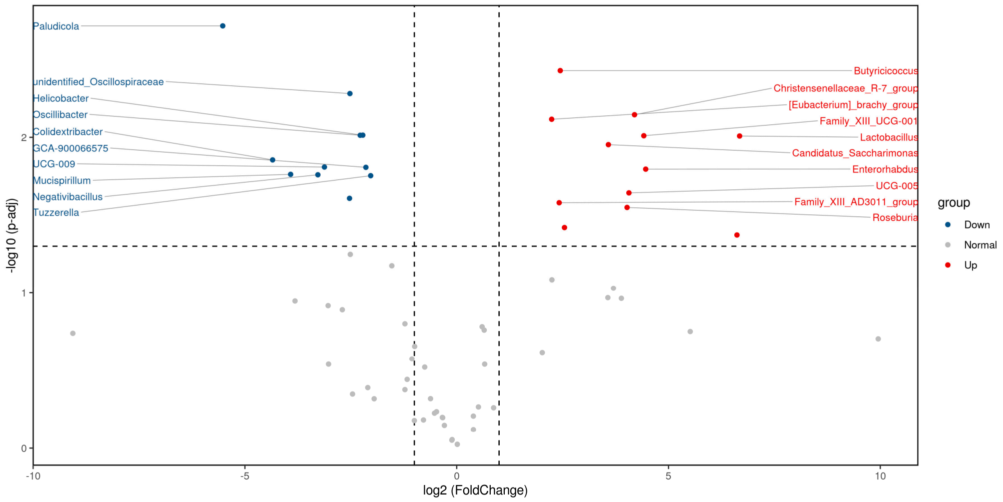

(**b**)

Supplementary Figure S1. (**a**) Differential abundance analysis of the genera between STD and MCD groups. Orange: Standart diet; Blue: Methionine-Choline-Deficient (MCD). Left Panel – Means in Groups: Horizontal bars represent the mean relative abundance of each genus in the two groups. Each taxon is listed on the Y-axis. The length of the bar reflects the average abundance level in each group. Right Panel – Difference Between Groups: Circles represent the mean difference in relative abundance between groups (STD - MCD). Horizontal error bars show the 95% confidence intervals. If the confidence interval does not cross zero, the difference is considered statistically significant. The dotted vertical line at 0 indicates no difference between groups. p-values: Displayed to the right of each row. Indicate the statistical significance of the difference between groups for each genus. Genera with $p < 0.05$ are considered significantly different. (**b**) Differential abundance analysis of the genera was carried out using ANCOM-BC for unpaired comparisons between STD vs. MCD groups Blue dots: most significantly overrepresented genera in the MCD group. Red dots: most significantly overrepresented genera in STD group.

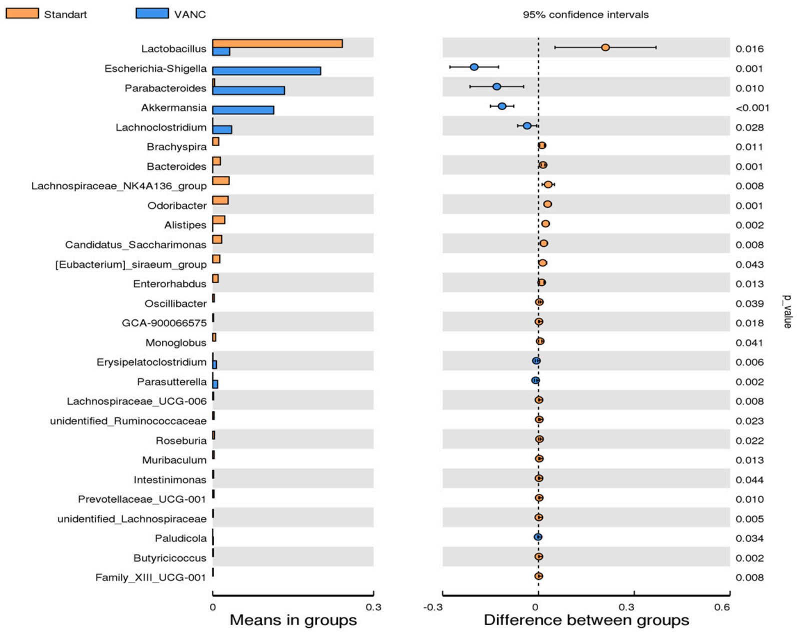

(**a**)

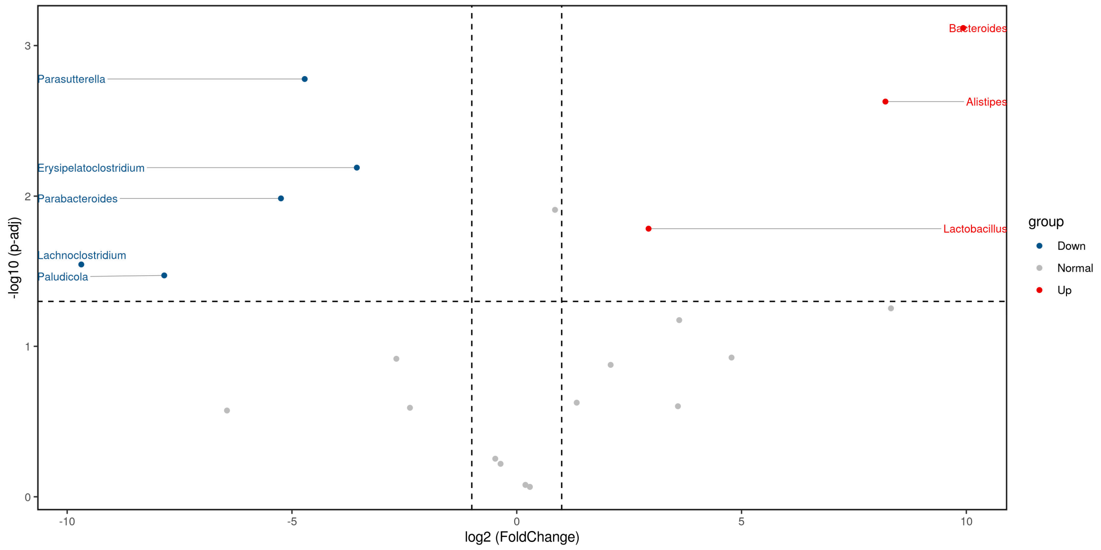

(**b**)

Supplementary Figure S2. (**a**) Differential abundance analysis of the genera between STD and VANC groups. Orange: Standart diet; Blue: Vancomycine (VANC). Left Panel – Means in Groups: Horizontal bars represent the mean relative abundance of each genus in the two groups. Each taxon is listed on the Y-axis. The length of the bar reflects the average abundance level in each group. Right Panel – Difference Between Groups: Circles represent the mean difference in relative abundance between groups (STD - VANC). Horizontal error bars show the 95% confidence intervals. If the confidence interval does not cross zero, the difference is considered statistically significant. The dotted vertical line at 0 indicates no difference between groups. p-values: Displayed to the right of each row. Indicate the statistical significance of the difference between groups for each genus. Genera with $p < 0.05$ are considered significantly different. (**b**) Differential abundance analysis of the genera was carried out using ANCOM-BC for unpaired comparisons between STD vs. VANC groups Blue dots: most significantly

overrepresented genera in the VANC group. Red dots: most significantly overrepresented genera in STD group.

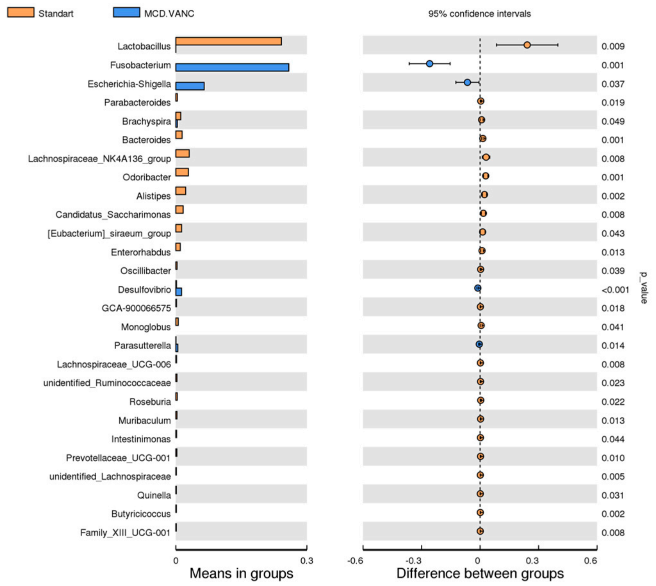

(**a**)

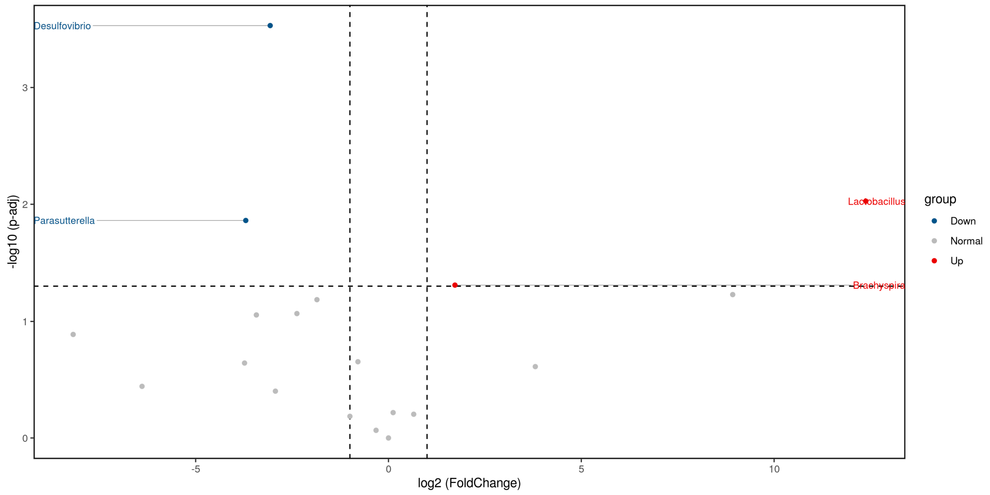

(**b**)

Supplementary Figure S3. (**a**) Differential abundance analysis of the genera between STD and MCD-VANC groups. Orange: Standart diet; Blue: Methionine-Choline-Deficient (MCD) diet and vancomycin. Left Panel – Means in Groups: Horizontal bars represent the mean relative abundance of each genus in the two groups. Each taxon is listed on the Y-axis. The length of the bar reflects the average abundance level in each group. Right Panel – Difference Between Groups: Circles represent the mean difference in relative abundance between groups (STD – MCD-VANC). Horizontal error bars show the 95% confidence intervals. If the confidence interval does not cross zero, the difference is considered statistically significant. The dotted vertical line at 0 indicates no difference between groups. p-values: Displayed to the right of each row. Indicate the statistical significance of the difference between groups for each genus. Genera with $p < 0.05$ are considered significantly different. (**b**) Differential abundance analysis of the genera was carried out using ANCOM-BC for unpaired comparisons between STD vs.

MCD-VANC groups Blue dots: most significantly overrepresented genera in the MCD-VANC group. Red dots: most significantly overrepresented genera in STD group.

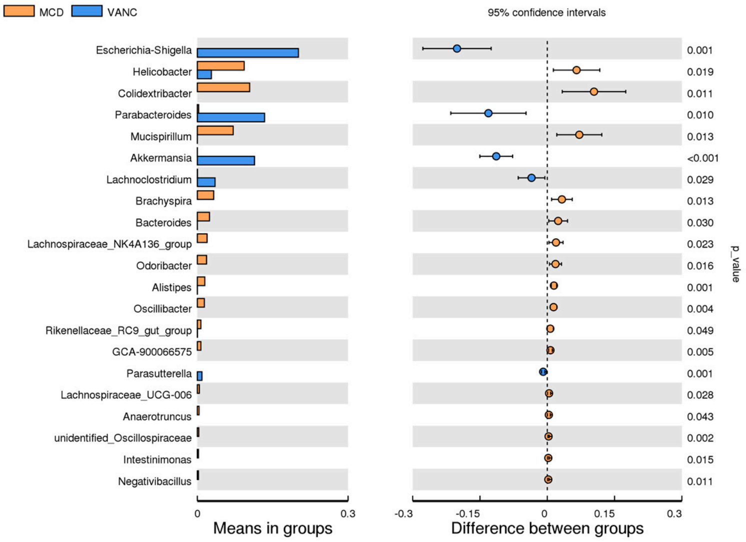

(a)

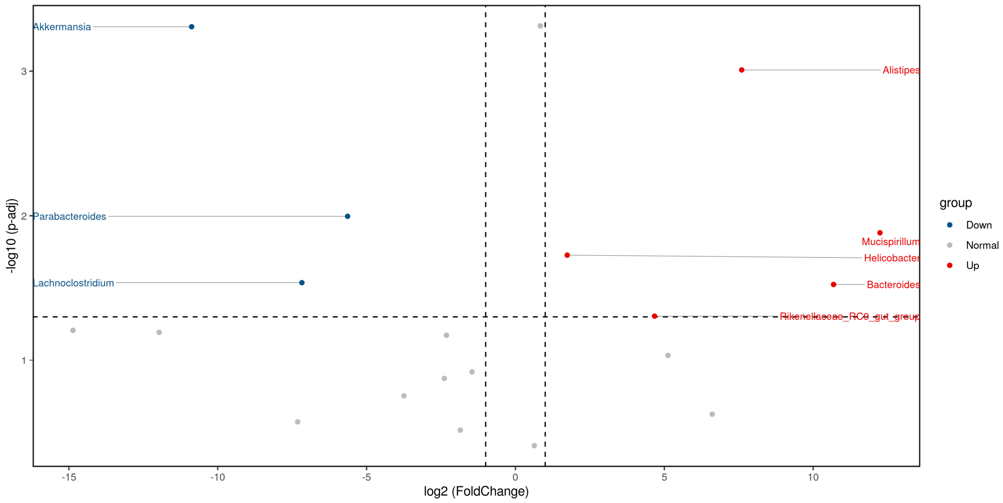

(b)

Supplementary Figure S4. (**a**) Differential abundance analysis of the genera between MCD and VANC groups. Orange: Methionine-Choline-Deficient (MCD) diet; Blue: vancomycin. Left Panel – Means in Groups: Horizontal bars represent the mean relative abundance of each genus in the two groups. Each taxon is listed on the Y-axis. The length of the bar reflects the average abundance level in each group. Right Panel – Difference Between Groups: Circles represent the mean difference in relative abundance between groups (MCD vs. VANC). Horizontal error bars show the 95% confidence intervals. If the confidence interval does not cross zero, the difference is considered statistically significant. The dotted vertical line at 0 indicates no difference between groups. p-values: Displayed to the right of each row. Indicate the statistical significance of the difference between groups for each genus. Genera with $p < 0.05$ are considered significantly different. (**b**) Differential abundance analysis of the genera was carried out using ANCOM-BC for unpaired comparisons between MCD vs. VANC groups Blue dots: most

significantly overrepresented genera in the VANC group. Red dots: most significantly overrepresented genera in MCD group.

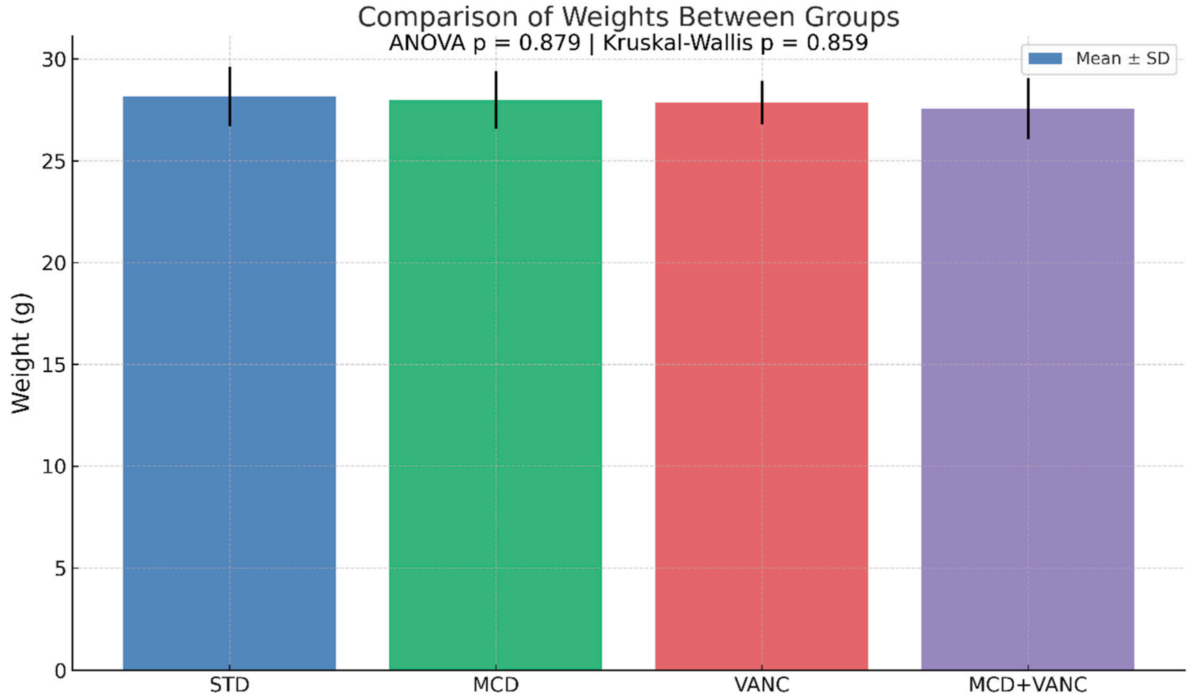

Supplementary Figure S5. Comparison of body weights across experimental groups. Mean ± SD body weight of mice in the standard diet (STD), methionine–choline-deficient diet (MCD), vancomycin-treated (VANC), and combined MCD+VANC groups after 10 weeks of intervention. No statistically significant differences in body weight were observed among groups (ANOVA $p = 0.879$; Kruskal–Wallis $p = 0.859$).
